# Isolation and Characterization of *Aeromonas salmonicida* Phage TSW001 and Its Application on Large Yellow Croaker

**DOI:** 10.3390/foods14122082

**Published:** 2025-06-12

**Authors:** Jun Yan, Zhenghao Guo, Jing Xie

**Affiliations:** 1 College of Food Science & Technology, Shanghai Ocean University, Shanghai 201306, China; 2 Laboratory for Quality and Safety Risk Assessment of Aquatic Products in Storage and Preservation of Ministry of Agriculture and Rural Affairs, Shanghai Ocean University, Shanghai 201306, China; 3 National Experimental Teaching Demonstration Center for Food Science and Engineering, Shanghai Ocean University, Shanghai 201306, China; 4 Shanghai Professional Technology Service Platform on Cold Chain Equipment Performance and Energy Saving Evaluation, Shanghai Ocean University, Shanghai 201306, China

**Keywords:** large yellow croaker, *Aeromonas salmonicida*, phage, biocontrol, biofilm

## Abstract

*Aeromonas salmonicida* is a common spoilage bacterium found in refrigerated fish. In this study, a virulent bacteriophage was isolated from wastewater using *A. salmonicida* AS08 as the host, and it was designated as TSW001. Based on morphological characterization and whole-genome analysis, bacteriophage TSW001 was classified within the genus *Tedavirus*. Biological characterization revealed that TSW001 maintained a stable titer within a temperature range of 4~60 °C, a pH range of 4~9, and a salinity range of 50~1000 mM. The optimal multiplicity of infection (MOI) for TSW001 was 0.1, with a short latency period of approximately 10 min and a burst size of approximately 68 PFU/cell. When applied during the cold storage of large yellow croaker, the *A. salmonicida* count in the fish juice decreased by approximately 2.1~2.3 log_10_ CFU/mL over the first two days, while the count in the fish fillets decreased by approximately 1.1~1.8 log_10_ CFU/g. Furthermore, TSW001 demonstrated the ability to inhibit the formation of *A. salmonicida* biofilms. These results suggest that phage TSW001 is a promising biological antimicrobial agent for controlling *A. salmonicida* during the cold storage of seafood.

## 1. Introduction

*Aeromonas salmonicida*, a Gram-negative bacillus belonging to the genus *Aeromonas*, is commonly found as a pathogen and organism in aquatic products. It is widely distributed and is a natural part of the microflora in refrigerated fish. Various aquatic products, including large yellow croaker, perch, and salmon, serve as hosts for this bacterium [1]. *A. salmonicida* is capable of secreting lipases and proteases, which promote the synthesis of amine metabolites, soften the surface of aquatic products, and produce mucus and off-flavors [2]. Furthermore, *A. salmonicida* is susceptible to forming biofilms on surfaces (e.g., plastic and stainless steel) in food processing contact surface environments [3]. Biofilms are complex microbial communities formed by bacterial cells that secrete extracellular substances themselves and encapsulate bacteria within them [4]. The presence of biofilms enhances their ability to withstand adverse environmental conditions, including antibiotics and disinfectants, making them challenging to eradicate. Additionally, biofilms may harbor resistance and virulence genes, leading to persistent contamination during processing and storage, resulting in significant economic losses [5], and statistically, more than 60% of foodborne illnesses are caused by biofilms [6].

In order to control the hazards brought by *A. salmonicida* to seafood, various methods have been studied and applied, including high-voltage electric fields, ultrasound, irradiation, antibiotics, and organic acids [7]. However, these methods have certain drawbacks, including high cost, the potential generation of drug-resistant bacteria, an unsatisfactory inhibition effect, and an unfavorable impact on the quality of seafood. In recent years, natural antimicrobial agents have been widely researched and applied in seafood preservation due to their environmentally friendly, non-toxic, and healthy properties [8]. Natural antimicrobial agents are mainly divided into four categories: plant-derived, animal-derived, microorganism-derived, and mineral-derived antimicrobials [9,10]. Among these, the discovery and application of phages have provided an effective weapon against the challenges of microorganisms and their biofilms [11].

Phages are viruses that can infect and kill specific bacteria. In practical applications, they help to minimize the side effects of antibiotics or other antibacterial chemicals on the environment, animals, and humans. Due to their safety, effectiveness, and low cost, significant progress has been made in their research in the food industry. A wide variety of phages have been isolated, characterized, and studied, with the objective of controlling foodborne pathogens (e.g., *Salmonella*, *Listeria monocytogenes*, and *Vibrio parahaemolyticus*) and spoilage bacteria (e.g., *Pseudomonas fluorescens*, *Shewanella putrefaciens*, and *A. salmonicida*) in food [12,13,14,15]. Moreover, bacteriophage-derived enzymes (such as tail lysins, endolysins, and polysaccharide depolymerases) have demonstrated efficacy in controlling bacterial growth and reducing bacterial colonization [16,17]. In particular, the discovery and application of depolymerases have provided an important weapon for biofilm prevention and control.

In this study, we isolated and identified a novel *A. salmonicida* phage, TSW001, from a food market in Shanghai, China, and investigated its morphological, biological, and genetic characteristics. Meanwhile, we evaluated the in vitro antibacterial effects of phage TSW001, its efficacy in preventing biofilm formation, and its ability to eradicate mature biofilms. In addition, the efficacy of phage TSW001 in food matrices was evaluated using juice and fillet of fresh large yellow croaker as models.

## 2. Materials and Methods

### 2.1. Bacterial Strains and Growth Conditions

*A. salmonicida* AS08, serving as the bacterial host for phage amplification in this research, was derived from a decayed large yellow croaker kept at 4 °C and has been assigned the GenBank accession number PP529988.1. The strain was cryopreserved at −80 °C in a 50% (*v*/*v*) glycerol solution and incubated in Luria–Bertani (LB) broth (Qingdao Hope Bio-Technology Co., Ltd., Qingdao, China) at 30 °C for a duration of 8 h.

### 2.2. Phage Isolation, Purification, and Enrichment

Wastewater samples were collected from a food market in Shanghai, China, and phages were isolated and purified using the method described by Guo et al. [14]. The wastewater sample was centrifuged at 5000× *g* for 10 min at 4 °C to remove solid debris, and the supernatant was filtered through a 0.22 μm membrane filter. The phage–bacteria mixture was added to 5 mL of molten LB top agar (0.5% agar), maintained at approximately 45 °C. This mixture was then poured evenly onto the surface of a pre-solidified LB agar plate (1.5% agar) and allowed to solidify at room temperature before incubation. The plates were incubated in an inverted position at 30 °C for 8 h. For phage purification, large and bright plaques were selected from the plate using an inoculation loop and transferred into 1 mL of SM buffer solution with thorough mixing. The purification process was repeated at least three times until the plaques exhibited uniform morphology and consistent size distribution.

### 2.3. Morphological Analysis via Transmission Electron Microscopy (TEM)

Phages were pre-treated according to the method of Guo et al. [14]. Imaging of the stained phage was conducted using a transmission electron microscope (Hitachi H-7650, Tokyo, Japan) operating at 80 kV.

### 2.4. Genomic DNA Isolation and Analysis of the Genome

Phage TSW001 genomic DNA was extracted using the phenol–chloroform method [10]. The extracted genomic DNA was sent to Shanghai Sangon Biotech for high-throughput sequencing analysis. In brief, the quality of raw Illumina sequencing data was assessed using FastQC, followed by quality trimming with Trimmomatic to obtain high-quality, clean reads. The filtered reads were then assembled using SPAdes with or without a reference genome. Assembly gaps were filled using GapFiller, and sequence errors as well as small insertions and deletions were corrected with PrInSeS-G. For genome annotation, Prokka was employed to predict genomic features including coding sequences, tRNA, and rRNA genes, while RepeatMasker was used to identify repetitive sequences. Functional annotation of predicted proteins was performed using NCBI Blast+ against multiple databases such as CDD, KOG, COG, NR, NT, PFAM, SwissProt, and TrEMBL. Gene Ontology (GO) terms were assigned based on annotations from SwissProt and TrEMBL, and KEGG pathway annotation was conducted using the KAAS platform.

### 2.5. Phage Lytic Range

Next, 2 μL of the diluted phage solution was dropped onto the bacterial plates listed in Table 1. After incubating overnight at 30 °C, the plates were observed to determine the presence of plaques on each plate.

### 2.6. Stability Tests of Bacteriophage TSW001

To assess thermal stability, phages (10^6^ PFU/mL) were exposed to various temperatures (−20 °C, 0 °C, 4 °C, 10 °C, 20 °C, 30 °C, 40 °C, and 50 °C) for 1 h, and their titers were determined using the double-layer agar plate method. For pH stability testing, LB culture medium was adjusted to different pH values (ranging from 2 to 10) using 1 M HCl or 1 M NaOH. Phages (10^6^ PFU/mL) were then subjected to one hour of treatment at 30 °C under various pH conditions, followed by determination of phage titers. To evaluate the salt stability of the bacteriophage, 100 μL of the phage suspension was mixed with 900 μL of NaCl solutions at varying concentrations (50 mM, 100 mM, 200 mM, 400 mM, 800 mM, and 1000 mM) [18]. The mixtures were incubated at 4 °C for 1 h, after which the phage titer was measured.

### 2.7. Multiplicity of Infection Evaluation

The multiplicity of infection (MOI) represents the ratio of phages to host strain cells during phage infection [11]. Phages were combined with the host strain at various MOI levels (10,000, 1000, 100, 10, 1, 0.1, and 0.01). The mixture was then incubated at 30 °C with shaking at 200 rpm for 5 h and subsequently centrifuged at 5000× *g* at 4 °C for 10 min. The phage titer in the resulting supernatant was determined to ascertain the optimal MOI.

### 2.8. One-Step Growth Assay of Phage TSW001

The one-step growth curve experiment was conducted following the method by Guo et al. [14] with some modifications. Briefly, 1 mL of logarithmic-phase host bacteria (OD600 ≈ 0.5, approximately 10^6^ CFU/mL) was mixed with an equal volume of bacteriophage suspension at a multiplicity of infection (MOI) of 0.1. The mixture was incubated at 30 °C for 5–10 min to facilitate phage adsorption, followed by centrifugation at 5000× *g* for 10 min to remove unbound phages. The resulting pellet was resuspended in 10 mL of fresh LB medium and incubated at 30 °C with continuous shaking. Aliquots were collected at regular intervals and immediately quantified using the double-layer agar method.

### 2.9. In Vitro Bactericidal Effects of TSW001

The *A. salmonicida* was cultured to the logarithmic phase (approximately 10^8^ CFU/mL) and then diluted 100-fold with fresh LB medium. A 100 μL aliquot of the bacterial suspension was added to each well of a 96-well plate, followed by an equal volume of bacteriophage suspension at different MOI levels (0.01, 0.1, 1, 10, 100, 1000, and 10,000). The plate was incubated with shaking at 30 °C, and the OD_600nm_ was measured every hour [14].

### 2.10. Effect of the Phage on A. salmonicida Biofilm

#### 2.10.1. Biofilm Formation and Quantification

The biofilm formation assay was performed as previously described with slight modifications [19]. The *A. salmonicida* was cultured to approximately 10^8^ CFU/mL (OD_600 nm_ ≈ 0.8) and then diluted 100-fold with fresh sterile LB medium. Stainless steel (diameter 12 mm, thickness 0.2 mm) was placed into 24-well polystyrene microtiter plates. The plates were incubated at 4 °C for 72 h without agitation to allow biofilm maturation. To evaluate the effect of phage TSW001 on biofilm formation, bacterial suspension and phage suspension (10^8^ PFU/mL) were simultaneously added to a 24-well plate and co-cultured with the host bacteria under static conditions at 4 °C for 72 h, followed by quantitative analysis. For mature biofilms, the phage suspension (10^8^ PFU/mL) was added after rinsing three times with sterile PBS buffer, followed by continued incubation for 4 h.

#### 2.10.2. Visualization by Confocal Laser Scanning Microscopy (CLSM)

The CLSM analysis followed the protocol of Yan and Xie [20]. After incubation as described in Section 2.10.1, the stainless steel sheets were washed twice with 0.1 M PBS buffer and then fixed with 4 % (*v*/*v*) glutaraldehyde at 4 °C for 1 h. After rinsing with 0.1 M PBS to remove the glutaraldehyde, the stainless steel sheets were stained with SYBR Green I in the dark for 15 min. Finally, images were captured using a confocal laser scanning microscope (LSM710, Carl Zeiss AG, Oberkochen, Germany) with a 40 × objective.

### 2.11. Effect of Phage TSW001 Against A. salmonicida on Large Yellow Croaker

Fresh large yellow croakers were purchased from a local market in Shanghai, China, and the internal organs, gills, and skin were removed. To prepare sterile fish juice, equal parts fish and ultrapure water were combined in a beaker, boiled for 15 min, then extracted, filtered, and sterilized using an autoclave (Henan Xinling Instrument & Equipment Co., Ltd., Jiaozuo, Henan, China) at 121 °C for 15 min. To prepare sterile fish fillets, the large yellow croaker was cut into 1 cm^2^ sections on a clean bench, treated with 75% ethanol for 5 min, rinsed twice with sterile water, and allowed to air dry under UV irradiation for 30 min. The fish juice and fish fillets were inoculated with serially diluted AS08 (~10^3^ CFU/mL), and phage TSW001 (~10^6^ PFU/mL) was subsequently added. All samples were stored at 4 °C for 4 days.

### 2.12. Statistical Analysis

Data analysis was conducted using SPSS software (version 17.0, SPSS Inc., Chicago, IL, USA). Variations among groups were evaluated through one-way analysis of variance (ANOVA), followed by Tukey’s post hoc test for pairwise comparisons. A *p*-value of less than 0.05 was considered statistically significant, whereas a threshold of *p* < 0.01 indicated highly significant differences.

## 3. Results

### 3.1. Isolation and Morphological Characterization of Phage TSW001

**Figure 1:**
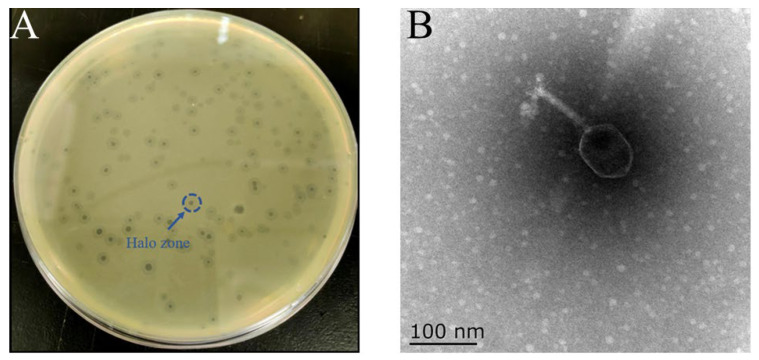
Morphology of the phage. (**A**) Plaque formation with a halo zone, highlighted by a blue circle, produced by TSW001 and (**B**) its morphology as observed via transmission electron microscopy. The scale bar represents a magnification of 100 nm.

A virulent phage, designated TSW001, was isolated from food market wastewater using *A. salmonicida* AS08 as the host bacterium. TSW001 formed transparent patches with a halo zone on the bacterial lawn of *A. salmonicida* using a double-layer agar method (Figure 1A). Purified phage particles displayed an icosahedral head with dimensions of 85.6 ± 9.6 nm in length and 65.8 ± 10.4 nm in width, along with a contractile tail measuring 94.8 ± 5.3 nm in length (Figure 1B).

### 3.2. Host Range of Phage TSW001

A total of 12 bacterial strains were selected to assess the host range of the bacteriophage, namely, 6 strains of *Aeromonas*, 3 strains of *Pseudomonas*, 1 strain of *Shewanella putrefaciens*, 1 strain of *Hafnia paralvei*, and 1 strain of *Acinetobacter johnsonii*. The findings, as shown in Table 1, reveal that bacteriophage TSW001 demonstrates lytic activity against *Aeromonas salmonicida* and *Aeromonas hydrophila*, while no lytic activity was observed against the other bacterial strains tested.

### 3.3. The Stability and Lysis Kinetics of Phage TSW001

**Figure 2:**
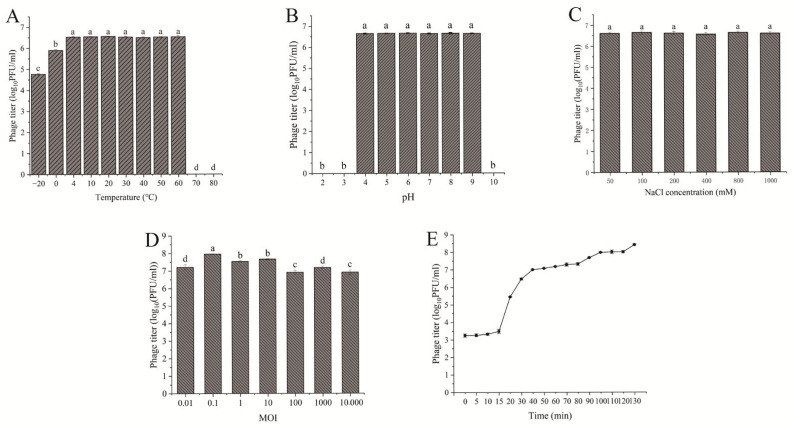
Characteristics of TSW001. The different letters represent significant differences (*p <* 0.05). (**A**) Stability of phage TSW001 at different temperatures. (**B**) Stability of phage TSW001 at different pH. (**C**) Stability of phage TSW001 at different NaCl concentration (**D**) Optimal multiplicity of infection (MOI) determination. (**E**) One-step growth curve.

Temperature and pH are crucial factors affecting phage activity. Therefore, the stability of the phage was assessed across different temperature and pH ranges. Statistical analysis revealed that phage TSW001 remained stable and maintained high activity within the temperature range of 4 °C to 60 °C, as shown in Figure 2A. The phage exhibited high stability between pH 4 and 9, but its stability was abruptly reduced under the extreme conditions of pH 2, 3, and 10 (Figure 2B). As illustrated in Figure 2C, phage TSW001 retains high activity across a sodium chloride concentration range of 50 to 1000 mM. The lysis capacity of a phage is closely related to its potential application. Phage TSW001 generated a maximum titer when infecting at an optimal MOI of 0.1 (Figure 2D). The one-step growth curve analysis revealed that phage TSW001 exhibited a latency phase of around 10 min and a burst size of approximately 68 PFU/cell, as illustrated in Figure 2E.

### 3.4. Genetic Characterization of TSW001

**Figure 3:**
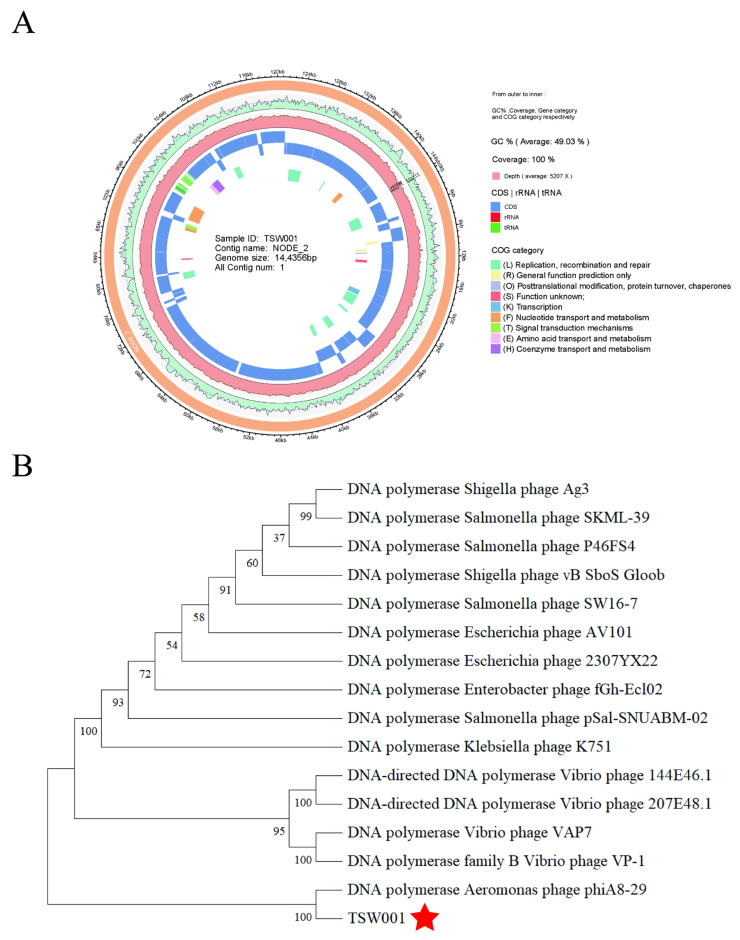
Genomic analysis of phage TSW001. (**A**) Genome map of TSW001; (**B**) phylogenetic tree based on DNA polymerase of 16 phages including bacteriophage TSW001. The tree was generated in Mega 7.0 using neighbor joining method with P distance values and 1000 bootstrap replicates. The asterisk (*) denotes the organism studied in our research.

The complete genome of phage TSW001 is 144,356 bp in length, with a G + C content of 49.03%. A total of 195 protein-coding genes were identified, accounting for 133,968 bp of the genome and representing 92.80% of the total genome length. Additionally, 14 tRNA genes were detected (Figure 3A). The predicted protein-coding sequences were annotated by BLASTP searches against the NR, Swiss-Prot, CDD, NOG, KEGG, and GO databases. A total of 53 protein-coding genes with known functions were identified in the TSW001 genome, which are primarily associated with structural components, DNA replication, and host cell lysis. In terms of structural components, the genome encodes a capsid protein gene (ORF 70), five tail-related genes (ORF 35, ORF 54, ORF 55, ORF 63, and ORF 64), a neck protein-related gene (ORF 60), a head-associated protein gene (ORF9), and two head–tail connector genes (ORF 50 and ORF165). Genes related to DNA replication include DNA polymerase II (ORF 3), DNA ligase (ORF 156), and an ATP-dependent DNA helicase (ORF 40). For applications in the food industry, it is essential that phages used as biocontrol agents do not harbor any virulence or antibiotic resistance genes. Phage lysis genes include endolysin-encoding genes (ORF 9 and ORF 13). Comparative analysis using the ARDB and VFDB databases confirmed that the TSW001 genome does not contain genes associated with antibiotic resistance, virulence factors, integrases, or allergenicity.

According to BLASTn analysis, the genome sequence of TSW001 shares a high degree of similarity with the published genome of A. phage phiA8-29, showing 70% query coverage and 81.67% sequence identity (GenBank accession number: KY914485). In the phylogenetic analyses (Figure 3B), TSW001 was clustered in the same branch with *Aeromonas* phage phiA8-29 in the genus *Tedavirus*. The genomic test data of phage TSW001 (GenBank accession number: PV714136) was registered in the NCBI database.

### 3.5. Phage Treatment Inhibits the Growth of Bacteria and Their Biofilms

**Figure 4:**
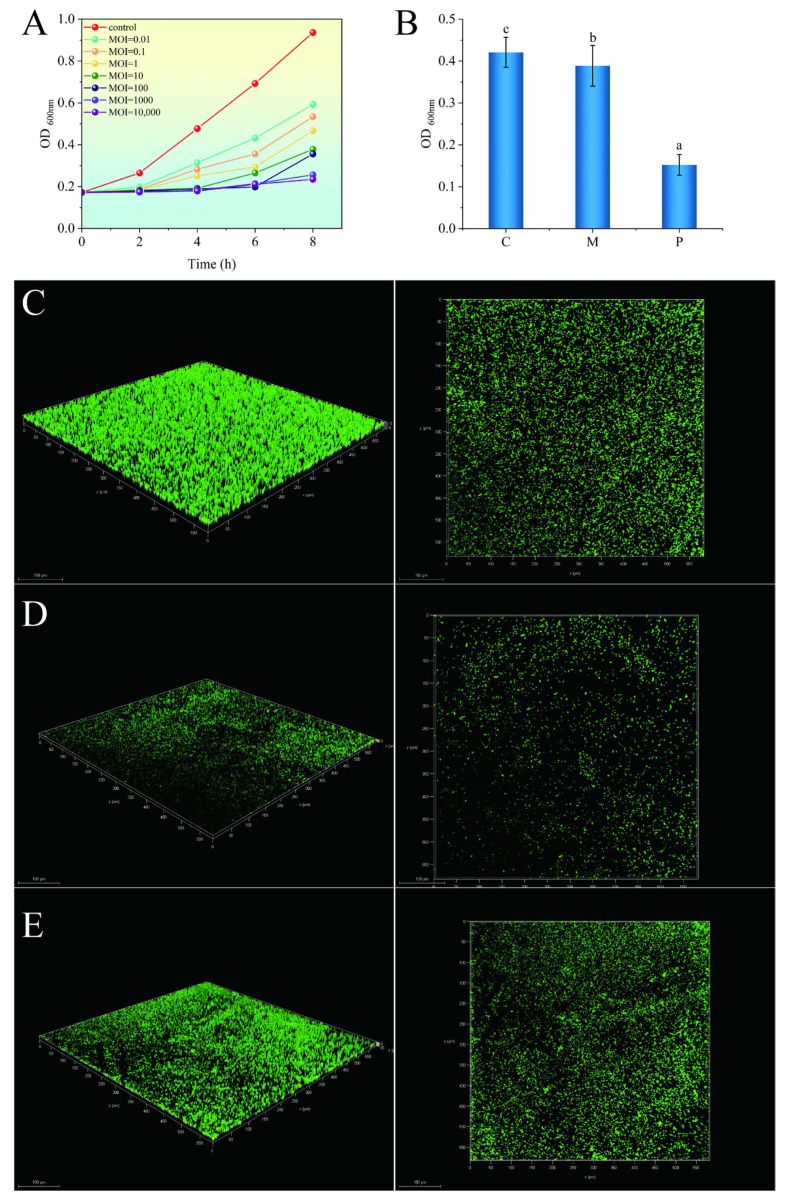
(**A**) Lysis activity of phage TSW001 under different MOI conditions; (**B**) biofilm quantification by crystal violet staining after treatment. C, M, P respectively represent the control group untreated with the phage; mature biofilm treated with the phage; developing biofilm treated with the phage. The different letters represent significant differences (*p* < 0.05). (**C**) CLSM images of mature biofilms not treated with phage TSW001; (**D**) CLSM images of the developing biofilm treated with phage TSW001; (**E**) CLSM images of the mature biofilm treated with phage TSW001.

The inhibitory effect of phage TSW001 on AS08 was evaluated at different multiplicity of infection levels. Compared to the control, treatment with the phage resulted in a significant reduction in the number of *A. salmonicida* counts at all MOIs (Figure 4A). Within the MOI range of 0.01 to 100, with the increase in the MOI, the inhibitory effect on *A. salmonicida* becomes more pronounced.

The effectiveness of phage TSW001 in inhibiting biofilm formation and eliminating mature biofilm was assessed on stainless steel, a material frequently employed in seafood processing equipment. Crystal violet staining showed that the TSW001 phage was not effective in removing mature biofilms but was highly effective in inhibiting biofilm formation, with a decrease in absorbance of approximately 0.27 at OD_600nm_ compared to the untreated control (Figure 4B). The 3D structure and thickness variations of biofilms on stainless steel sheets were evaluated using CLSM analysis. After 72 h at 4 °C, the control group, which was not treated with the phage, formed a densely structured and compact biofilm (Figure 4C). A sparse and dispersed distribution throughout the biofilm was observed when the developing biofilm was treated with the phage (Figure 4D). However, the structure of the mature biofilm did not change significantly and remained intact after treatment with the phage for 4 h (Figure 4E).

### 3.6. Antibacterial Activity of Phage TSW001 Against AS08 in Large Yellow Croaker

**Figure 5:**
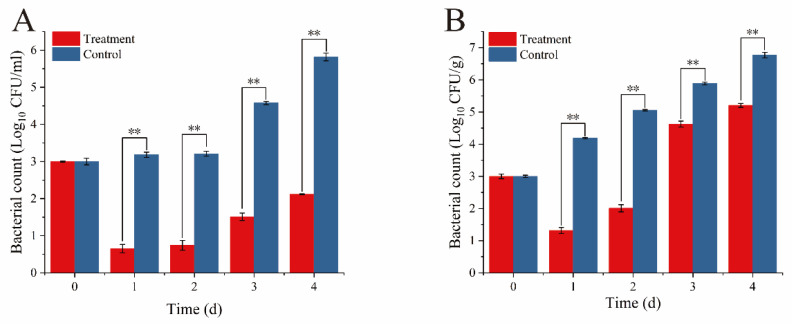
Antibacterial activity of phage TSW001 against AS08 in large yellow croaker. (**A**) Phage TSW001 for use in sterile fish juice; (**B**) phage TSW001 for use in sterile fish fillets. **: *p* < 0.01.

The results of the in vitro lysis experiments indicated that the phage exhibited the most pronounced inhibitory effect at MOI values of 1000 and 10,000. Therefore, an MOI of 1000 was selected for the evaluation of the effect of phage TSW001 in the food matrix. The number of AS08 decreased by approximately 2.1–2.3 log_10_ CFU/mL in the first two days in fish juice at 4 °C, but the number of AS08 increased significantly after the third day (Figure 5A). The inhibitory effect of fish fillets on AS08 was slightly less pronounced than that of fish juice, with a reduction of approximately 1.1–1.8 log_10_ CFU/g over the first two days. However, there was also a significant increase in the number of AS08 on day 3 compared to the previous two days (Figure 5B).

## 4. Discussion

Phages and their derived enzymes demonstrate potential as antimicrobial agents, exhibiting natural bioinhibitory properties with potent bacteriostatic activity and biofilm control. Their utilization for managing detrimental microbial contamination in food and processing environments is steadily increasing [21,22]. In this study, a novel *A. salmonicida* phage was isolated and characterized from the effluent of a food market, designated as TSW001. Phage TSW001 can form plaque with a halo zone on LB solid medium plates, suggesting its potential to produce depolymerases (Figure 1A). Morphological analysis revealed that the phage consists of an icosahedral head and a flexible tail, which belongs to the *Ackermannviridae* family. Furthermore, the one-step growth curve of phage TSW001 demonstrated a high burst size and a short latency, indicating that the phage ’s life cycle is suitable for biocontrol. This is because the phage can spread rapidly and kill bacterial populations.

The circumstances of seafood processing are complex and variable, and the phage must have good environmental tolerance to perform its biological activity optimally. In the actual production process, the large yellow croaker is typically refrigerated at temperatures between 0 °C and 4 °C, and the pH during storage is usually between 6 and 8 [23]. The experimental results demonstrate that bacteriophage TSW001 maintained a stable titer within the temperature range of 4–60 °C. However, following incubation at −20 °C for 1 h, the phage titer exhibited a moderate reduction of approximately 2 log_10_ PFU/mL. Complete inactivation occurred after 1 h of incubation at 70 °C and 80 °C. Furthermore, TSW001 remained stable across a pH range of 4–9 and a salinity range of 50 mM–1000 mM. The temperature and pH stabilization range of TSW001 is comparable to that of other *A. salmonicida* phages, as evidenced by previous studies [24,25].

The practical applicability of a phage is determined by its safety and bacteriostatic properties. The analysis of the genomic DNA revealed that phage TSW001 does not possess genes associated with antibiotic resistance, toxins, lysozymes, or virulence factors. This indicates that it is biologically safe for use in food applications. At the same time, the phage may encode multiple caudal fibrillar proteins (ORF 35, 54, 55) that aid in the efficient recognition of host surface receptors, potentially reducing the incubation period. Structural domain predictions also suggest the presence of the pectin lysis enzyme structural domain in tail fibronectins (ORF 54, 55), indicating the ability to target live cells within biofilms while unaffected by EPS. Moreover, phage TSW001 carries genes for various lytic proteins, including endolysin (ORF 9, 13) and tail lysozyme (ORF 7, 8), which are critical for processes such as adsorption, invasion, lysis, and biofilm elimination.

The formation of biofilms is influenced by a multitude of environmental factors, including temperature, humidity, and osmotic pressure, as well as the characteristics of the attachment surface and the bacterial strain. This study demonstrated that the *A. salmonicida* strain was capable of forming a dense biofilm in a simulated processing environment. The utilization of lytic phages and their enzymes has emerged as a promising method for disrupting biofilm integrity in the food industry [26]. Although TSW001 was effective in preventing the formation of *A. salmonicida* biofilms, its ability to eliminate mature biofilms was limited. This discrepancy may be attributed to the heightened contact between phages and bacteria during the initial stages of biofilm formation, which results in more favorable infection outcomes [27]. When using phages to treat mature biofilms, the movement of the phages within the biofilm is restricted [28], and phages can be inactivated by amidases and peptidases present in the biofilm matrix [29]. In addition, although phage TSW001 is capable of encoding depolymerase to overcome the restriction of EPS in the biofilm, phages can also bind to dead cells, proteins, lipopolysaccharides, and other components in the biofilm, which limits the phage infection of living cells [30]. Conversely, the efficacy of phage lysis is diminished by nutrient scarcity in the environment, which impairs host cell growth and metabolism [31]. Consequently, the removal of mature biofilms is less successful.

The growth and metabolism of *A. salmonicida* can lead to the accumulation of trimethylamine (TMA), cadaverine, and total volatile basic nitrogen (TVB-N), resulting in a fishy odor in large yellow croaker [3]. The application of TSW001 to fish fillets and juice effectively inhibited the growth of *A. salmonicida* within 48 h. However, a notable rebound in bacterial counts was observed after 48 h, which may be attributed to the emergence of phage-resistant mutants, phage inactivation by components in the fish matrix, or an insufficient initial phage dose. Following this period, the growth of *A. salmonicida* in the phage-treated group was significantly lower compared to the control group in both matrices. The treatment outcome was more effective in fish juice than in fish fillets, likely due to the restriction of phage movement in fish fillets. In addition, it is possible that the higher nutrient content in the fish fillets accelerates the growth of AS08, resulting in the antibacterial effect of TSW001 being less noticeable.

## 5. Conclusions

In this study, we isolated a virulent *A. salmonicida* phage, TSW001, which exhibits stability across a broad range of temperatures and pH levels. Genomic analysis revealed that the genome of phage TSW001 does not contain virulence or antibiotic resistance genes, indicating its biosafety. Phage TSW001 effectively inhibits biofilm formation and demonstrates a certain degree of efficacy in disrupting mature biofilms. In addition, phage TSW001 can better control *A. salmonicida*-induced spoilage in large yellow croaker. These results indicate that phage TSW001 can be used as an effective biological control agent for the prevention and control of seafood spoilage caused by specific spoilage organisms.

## Figures and Tables

**Table 1:** Host range of phage TSW001.

Strain	Source	Lytic
*A. salmonicida* AS03	*Oreochromis niloticus*	+
*A. salmonicida* AS08	*Larimichthys crocea*	+
*Aeromonas hydrophila*	*Oreochromis niloticus*	+
*Aeromonas sobria*	*Oreochromis niloticus*	−
*Aeromonas piscicola*	*Oreochromis niloticus*	−
*Pseudomonas mevalonii*	*Grouper*	−
*Acinetobacter johnsonii*	*Thunnus obesus*	−
*Shewanella putrefaciens*	*Thunnus obesus*	−
*Hafnia paralvei*	*Grouper*	−
*A. allosaccharophila*	*Grouper*	−
*Pseudomonas fluorescens*	*Larimichthys crocea*	−
*Pseudomonas fluorescens*	Milk	−

## Data Availability

The original contributions presented in the study are included in the article, further inquiries can be directed to the corresponding author.

## References

[1] Zhang C., Liu X., Li H., Hu T., Jatt A.-N., Liu Y. (2024). Antibacterial mechanism of lactobionic acid against *Aeromonas salmonicida* by transcriptomic analysis and its application in refrigerated grass carp. Food Control.

[2] Shao L., Dong Y., Chen S., Sheng J., Cai L., Xu X., Wang H. (2024). Revealing extracellular protein profile and excavating spoilage-related proteases of *Aeromonas salmonicida* based on multi-omics investigation. Int. J. Biol. Macromol..

[3] Liu L., Yan Y., Feng L., Zhu J. (2018). Quorum sensing asaI mutants affect spoilage phenotypes, motility, and biofilm formation in a marine fish isolate of *Aeromonas salmonicida*. Food Microbiol..

[4] Karygianni L., Ren Z., Koo H., Thurnheer T. (2020). Biofilm matrixome: Extracellular components in structured microbial communities. Trends Microbiol..

[5] Sharan M., Vijay D., Dhaka P., Bedi J.S., Gill J.P.S. (2022). Biofilms as a microbial hazard in the food industry: A scoping review. J. Appl. Microbiol..

[6] Tian Y., Tian X., Li T., Wang W. (2023). Overview of the effects and mechanisms of NO and its donors on biofilms. Crit. Rev. Food Sci. Nutr..

[7] Ling X.-D., Lv J., Chen F.-J., Qin X.-T., Wu M.-S., Bai F., Luo H.-Q. (2024). Expression characteristics and in vitro antibacterial properties of C-type lysozyme in crucian carp infected with *Aeromonas salmonicida*. Heliyon.

[8] Yan J., Guo Z., Zhao Z., Yuan J., Wang X., Xie J. (2024). High-value development and utilization of functional peptides from seafood by-products and discards: A case study of antimicrobial peptides. Food Biosci..

[9] Esmaeili Y., Paidari S., Baghbaderani S.A., Nateghi L., Al-Hassan A.A., Ariffin F. (2022). Essential oils as natural antimicrobial agents in postharvest treatments of fruits and vegetables: A review. Food Meas..

[10] Papadochristopoulos A., Kerry J.P., Fegan N., Burgess C.M., Duffy G. (2021). Natural Anti-Microbials for Enhanced Microbial Safety and Shelf-Life of Processed Packaged Meat. Foods.

[11] Kim B.H., Ashrafudoulla M., Shaila S., Park H.J., Sul J.D., Park S.H., Ha S.-D. (2024). Isolation, characterization, and application of bacteriophage on Vibrio parahaemolyticus biofilm to control seafood contamination. Int. J. Antimicrob. Agents.

[12] Kim S.-H., Lee H., Park M.-K. (2023). Isolation, characterization, and application of a novel, lytic phage vB_SalA_KFSST3 with depolymerase for the control of Salmonella and its biofilm on cantaloupe under cold temperature. Food Res. Int..

[13] Namonyo S., Weynberg K.D., Guo J., Carvalho G. (2023). The effectiveness and role of phages in the disruption and inactivation of clinical *P. aeruginosa* biofilms. Environ. Res..

[14] Guo Y., Li J., Islam M.S., Yan T., Zhou Y., Liang L., Connerton I.F., Deng K., Li J. (2021). Application of a novel phage vB_SalS-LPSTLL for the biological control of Salmonella in foods. Food Res. Int..

[15] Ning Z., Zhang L., Cai L., Xu X., Chen Y., Wang H. (2023). Biofilm removal mediated by Salmonella phages from chicken-related sources. Food Sci. Hum. Wellness.

[16] Azeredo J., Garcia P., Drulis-Kawa Z. (2021). Targeting biofilms using phages and their enzymes. Curr. Opin. Biotechnol..

[17] Górski A., Międzybrodzki R., Węgrzyn G., Jończyk-Matysiak E., Borysowski J., Weber-Dąbrowska B. (2020). Phage therapy: Current status and perspectives. Med. Res. Rev..

[18] Xiao K., Pan Q., Wu Y., Ding Y., Wu Q., Zhang J., Wang Z., Liu Z., Wang W., Wang J. (2025). Application of a novel phage vB_CjeM_WX1 to control *Campylobacter jejuni* in foods. Int. J. Food Microbiol..

[19] Yan J., Xie J. (2021). Removal of *Shewanella putrefaciens* Biofilm by acidic electrolyzed water on food contact surfaces. LWT.

[20] Yan J., Xie J. (2020). Comparative proteome analysis of *Shewanella putrefaciens* WS13 mature biofilm under cold stress. Front. Microbiol..

[21] López-Cuevas O., Medrano-Félix J.A., Campo N.C.-D., Chaidez C. (2021). Bacteriophage applications for fresh produce food safety. Int. J. Environ. Health Res..

[22] Vikram A., Callahan M.T.L., Woolston J.W., Sharma M., Sulakvelidze A. (2022). Phage biocontrol for reducing bacterial foodborne pathogens in produce and other foods. Curr. Opin. Biotechnol..

[23] Yan Q., Guo M., Chen B., Zhang C., Li D., Xie J. (2023). Molecular characterization of spoilage microbiota in high CO_2_ refrigerated large yellow croaker (*Larimichthys crocea*) fillets using metagenomic and metabolomic approaches. Food Biosci..

[24] Hosseini N., Paquet V.E., Marcoux P.-É., Alain C.-A., Paquet M.F., Moineau S., Charette S.J. (2023). MQM1, a bacteriophage infecting strains of *Aeromonas salmonicida* subspecies salmonicida carrying Prophage 3. Virus Res..

[25] Xu Z., Jin P., Zhou X., Zhang Y., Wang Q., Liu X., Shao S., Liu Q. (2021). Isolation of a virulent *Aeromonas salmonicida* subsp. masoucida bacteriophage and its application in phage therapy in turbot (*Scophthalmus maximus*). Appl. Environ. Microbiol..

[26] Mevo S.I.U., Ashrafudoulla M., Mizan M.F.R., Park S.H., Ha S.-D. (2021). Promising strategies to control persistent enemies: Some new technologies to combat biofilm in the food industry—A review. Compr. Rev. Food Sci. Food Saf..

[27] Pires D.P., Melo L.D.R., Boas D.V., Sillankorva S., Azeredo J. (2017). Phage therapy as an alternative or complementary strategy to prevent and control biofilm-related infections. Curr. Opin. Microbiol..

[28] Hu J., Miyanaga K., Tanji Y. (2010). Diffusion properties of bacteriophages through agarose gel membrane. Biotechnol. Prog..

[29] Flemming H.-C., van Hullebusch E.D., Neu T.R., Nielsen P.H., Seviour T., Stoodley P., Wingender J., Wuertz S. (2023). The biofilm matrix: Multitasking in a shared space. Nat. Rev. Microbiol..

[30] Drulis-Kawa Z., Majkowska-Skrobek G., Maciejewska B. (2015). Bacteriophages and phage-derived proteins—Application approaches. Curr. Med. Chem..

[31] Bryan D., El-Shibiny A., Hobbs Z., Porter J., Kutter E.M. (2016). Bacteriophage T4 infection of stationary phase *E. coli*: Life after log from a phage perspective. Front. Microbiol..

